# Efficiency of Biosynthesized Silver and Zinc Nanoparticles Against Multi-Drug Resistant Pathogens

**DOI:** 10.3389/fmicb.2018.02207

**Published:** 2018-09-20

**Authors:** Kapil Punjabi, Sourabh Mehta, Rujuta Chavan, Vidushi Chitalia, Dhanashree Deogharkar, Sunita Deshpande

**Affiliations:** ^1^Department of Clinical Pathology, Haffkine Institute for Training, Research and Testing, Mumbai, India; ^2^National Centre for Nanoscience and Nanotechnology, University of Mumbai, Mumbai, India; ^3^Department of Microbiology, Guru Nanak Khalsa College, Mumbai, India

**Keywords:** silver nanoparticles, zinc nanoparticles, *Pseudomonas hibiscicola*, MRSA, VRE, MDR-TB

## Abstract

Biosynthesis of metallic nanoparticles has acquired particular attention due to its economic feasibility, low toxicity, and simplicity of the process. In this study, extracellular synthesis of silver and zinc nanoparticle was carried out by *Pseudomonas hibiscicola* isolated from the effluent of an electroplating industry in Mumbai. Characterization studies revealed synthesis of 40 and 60 nm nanoparticles of silver (AgNP) and zinc (ZnNP), respectively, with distinct morphology as observed in TEM and its crystalline nature confirmed by XRD. DLS, zeta potential, NTA, and FTIR studies further characterized nanoparticles giving data about its size, stability, and functional groups. Considering the toxicity of nanoparticles the evaluation of antimicrobial activity was studied in the range of non-toxic concentration for normal cell lines. Silver nanoparticles were found to be the most effective antimicrobial against all tested strains and drug-resistant clinical isolates of MRSA, VRE, ESBL, MDR, *Pseudomonas aeruginosa* with MIC in the range of 1.25–5 mg/ml. Zinc nanoparticles were found to be specifically active against Gram-positive bacteria like *Staphylococcus aureus* including its drug-resistant variant MRSA. Both AgNP and ZnNP were found to be effective against *Mycobacterium tuberculosis* and its MDR strain with MIC of 1.25 mg/ml. The synergistic action of nanoparticles assessed in combination with a common antibiotic gentamicin (590 μg/mg) used for the treatment of various bacterial infections by Checker board assay. Silver nanoparticles profoundly exhibited synergistic antimicrobial activity against drug-resistant strains of MRSA, ESBL, VRE, and MDR *P. aeruginosa* while ZnNP were found to give synergism with gentamicin only against MRSA. The MRSA, ESBL, and *P. aeruginosa* strains exhibited MIC of 2.5 mg/ml except VRE which was 10 mg/ml for both AgNPs and ZnNPs. These results prove the great antimicrobial potential of AgNP and ZnNP against drug-resistant strains of community and hospital-acquired infections and opens a new arena of antimicrobials for treatment, supplementary prophylaxis, and prevention therapy.

## Introduction

New-age researchers are dedicated to upgrading the current world to a better and easier habitation. To achieve this, nanotechnology will play an important role in newer and improved materials that are robust, economic, and sources of renewable energy and pollution-free environment. The future doctors will use devices made up of such enhanced materials to not only ease the process of diagnostics but also considerably reduce the time and help treatment at early stages. Simpler and faster diagnosis coupled with efficient and reliable treatments will help to cure cancer, diabetes, and various infections. This may seem easy but a difficult road-map lies in front of these researchers, but certainly leads with help of nanotechnology makes it look possible and a revolution seems to be around the corner (Bhattacharyya et al., 2009).

With many arms of nanotechnology being shaped, one major domain is the biomedical application. The biomedical applications of nanotechnology comprise of many sub areas predominantly therapeutic and diagnostic areas have received considerable attention. Different nanoparticles and nanomaterials owing to their small size can easily interact with biomolecules at cellular as well as sub cellular levels. This greatly enhances the signaling of markers in case of diagnostics and improved specificity for targeted therapeutics (Conde et al., 2012).

Multiple applications of nanotechnology across fields like medical, chemical, environmental, agricultural, industrial, energy sciences, and information technology. This attracts major attention from various organizations in both developed and developing nations throughout the world (Roco, 2007).

Many approaches for the synthesis of nanoparticles have been proposed principally categorized into three classes namely – physical, chemical, and biological. Both physical and chemical methods are the major source of nanoparticles in the current period. While these methods ensure continuous supply they also have price to pay in terms of high energy requirements, toxic by-products. This highlights the requirement of sustainable and eco-friendly mode of synthesis. This brings into picture the biological methods with microorganisms playing a significant role. Thus, microbiologists around the world try to identify microorganisms for this capability and develop a simple and cost-effective method to synthesize nanoparticles of constant size, shape, and mono-dispersity (Babu et al., 2011; Horikoshi and Serpone, 2013).

Biosynthesized nanoparticles are ideal candidates for medical applications as they are biocompatible and naturally stable. One of the important property of such nanoparticles is antimicrobial activity. Many green silver nanoparticles have been reported for antibacterial and antibiofilm activity through mechanisms like ROS generation (Qayyum et al., 2017). Also supportive studies on plant derived silver nanoparticles conclude that biogenic nanomaterials are biocompatible and an effective therapeutic agent against bacterial, fungal infections, and cancer treatment (Oves et al., 2018).

Although nanoparticles are considered as future to many areas of science and technology, its probable environmental toxicity is a matter of concern. Thus, in this study, we synthesized nanoparticle, using bacteria isolated from a challenged environment like soil and effluent of electroplating industry and characterized to check potential therapeutic applications if rendered usable by evaluation of its *in vitro* toxicity.

## Materials and Methods

### Synthesis and Characterization of Nanoparticles

The synthesis and characterization of AgNP was followed by our previously reported work using the bacterial culture of *Pseudomonas hibiscicola* isolated from effluent of an electroplating industry. The culture inoculated in sterile nutrient broth and incubated at 37°C for 24 h in an orbital shaker. After incubation, it was centrifuged till clear supernatant was achieved. The supernatant was subjected to 1 mM concentration of silver nitrate AgNO3 (Sigma-Aldrich, United States) and incubated on a shaker with similar conditions. The bio-reduction of silver ions was monitored by visual observation and measuring absorption spectrum of samples using UV–vis Spectrophotometer Thermo scientific Nanodrop – 1000 v3.7 scanning range from 200 to 800 nm (Punjabi et al., 2017). For synthesis of ZnNP, similar procedure was followed; briefly the culture supernatant of *P. hibiscicola* was challenged with 10 mM zinc acetate [Zn(O_2_CCH_3_)_2_] (Himedia labs Pvt. Ltd., India) in 1:2 v/v ratio and incubated in similar conditions. After 24 h of incubation, precipitate formation and turbidity indicated presence of ZnNP in the reaction mixture. Reduction of metal ion to nanoparticle was confirmed by observing plasmon peak in the range of 320–380 nm in UV–Vis spectra (Waghmare et al., 2011).

To determine the size, morphology, and other characters of synthesized ZnNPs, characterization studies were undertaken. Suspension of zinc nanoparticles was subjected to UV–Vis spectroscopy, nanoparticle tracking, and analysis (NTA), DLS – particle size analyzer, and zeta potential analysis of the liquid suspension was done using NanoPlus DLS (Micromeritics Instrument Corporation). While powdered material was used for X-ray diffraction **(**XRD) patterns that were recorded on normal focus diffractometer (Regaku Miniflex, Japan) at voltage of 30 kV and current 15 mA with scan rate of 3°/min. The data were recorded in the range of 20°–50° and analyzed using Jade 6.0 software. Fourier transform infrared (FTIR) spectra obtained on a JASCO spectrometer (6100 type-A) instrument in the range of 400–4000 cm^−1^ were identified spectroscopy and TEM analysis on a Philips CEM 200 Microscope (Pattanayak, 2013).

### Toxicity of Nanoparticles

Toxicity of nanoparticles was assessed on Vero cell line (Monkey kidney cell line). The cell line, Vero passage number 27 was obtained from National Centre for Cell Sciences, Pune, India, in Dulbecco’s Modified Essential Medium (DMEM) supplemented with 10% fetal bovine serum (FBS) (complete medium).

To evaluate the effect of AgNP and ZnNP on cell toxicity colorimetric MTT (3-(4, 5-dimethylthiazol-2)-2, 5 diphenyl tetrazolium bromide) assay was performed. The MTT assay is a colorimetric technique wherein amount of absorbance is directly proportional to number of living cells. The MTT tetrazolium compound is reduced by viable cells into an intensely colored formazan precipitate that subsequently is solubilized into a uniformly colored solution with a second procedural step before absorbance is measured using a plate reading spectrophotometer (Freshney, 2010).

Known number (1 × 10^4^ cells) of cells was seeded into tissue culture grade 96-well plates (Riss et al., 2011). Cells were treated with various concentrations (10.0–0.6 mg/ml) of nanoparticles for 24 h and treated with 0. 5% MTT (HiMedia Laboratories Pvt. Ltd., India).

After 4 h, all the contents of wells were removed and 100 μl of dimethyl sulfoxide (DMSO) was added. The absorbance of colored solution was measured on the Multimode reader (Synergy HTX Multimode reader, BIOTEK, India) using a test wavelength of 490 nm (Freshney, 2010; Satyavani et al., 2011). Percentage toxicity was estimated for each concentration, and the IC_50_ value was calculated for both nanoparticles (Freshney, 2005).

### Antimicrobial Activity of Nanoparticles

#### Bacterial Strains

##### Gram-negative bacteria

*Proteus mirabilis* ATCC 21100, *Escherichia coli* ATCC 25922, *Klebsiella pneumoniae* ATCC 10031, *K. pneumoniae* ATCC 700603 (extended spectrum β lactamases producer, ESBL), *Pseudomonas aeruginosa* ATCC 27853, *Enterobacter aerogenes* ATCC 13048, *Salmonella typhimurium* ATCC 14028, *Salmonella abony* ATCC 6017, and *Shigella boydii* ATCC 8700.

##### Gram-positive bacteria

*Staphylococcus aureus* ATCC 25923, *Bacillus subtillis* ATCC 6633, *S. aureus* ATCC 43300 (methicillin-resistant *S. aureus*, MRSA), *S. aureus* ATCC 6538 *Enterococcus faecalis* ATCC 51299 (vancomycin-resistant *Enterococci*, VRE), *Staphylococcus epidermidis* ATCC 12228, and *Bacillus cereus* ATCC 11778.

##### Mycobacteria

*Mycobacterium tuberculosis* H37Rv.

##### Clinical strains

MDR *P. aeruginosa* and MDR *M. tuberculosis.*

ATCC strains were procured from Clinical Pathology department, Haffkine Institute, microbial culture collection (MCC) Pune, India, and Himedia laboratories Pvt. Ltd., Mumbai, India. The MDR strains of clinical isolates were procured from tertiary care hospital in Mumbai. The drug resistance of the isolates was confirmed before use in the study as per standard protocols of Clinical and Laboratory Standards Institute [CLSI] (2012).

#### Minimum Inhibitory Concentration

Antimicrobial activity of the nanoparticles synthesized was determined by broth dilution method and confirmation by growth on solid media (Wiegand et al., 2008; Ataee et al., 2011). For determining the minimum concentration of nanoparticles required for the inhibition of bacterial growth, broth dilution method was used according to standard broth microdilution method M07 A9 of Clinical and Laboratory Standards Institute [CLSI] (2012). This method facilitates the testing of inhibitory activity at various nanoparticle concentrations (Clinical and Laboratory Standards Institute [CLSI], 2012).

#### Anti-mycobacterial Activity – Broth Dilution Method

The minimum inhibitory concentrations (MICs) of silver and zinc nanoparticles were assessed by broth dilution method against *M. tuberculosis* H37Rv and MDR strain. Tenfold serial dilutions of the inoculum suspension were made in M7H9 broth supplemented with Middlebrook OADC enrichment. The stock concentrations of nanoparticles were serially diluted to obtain concentrations in the range 1.25–10 mg/ml. The prepared bacilli suspension (500 μl) was added to all tubes except the control tubes, all the tubes were incubated at 37°C for 7 days, which is physiological temperature favorable for growth of pathogenic strains. To determine the MIC, 10 μl of each tube was transferred to Lowenstein Jensen Medium (LJ medium) and incubated at 37°C for 3 weeks. After incubation, growth on LJ media was analyzed for each concentration of both nanoparticles. The observations were recorded and MIC values were determined (Andrews, 2001; Jadaun et al., 2007).

### Synergistic Activity of Gentamicin and Nanoparticles

#### Disk Diffusion Test for Gentamicin

Antibiotic susceptibility test (AST) against gentamicin (10 μg) and high-level gentamicin (120 μg) (HiMedia labs Pvt. Ltd.) was performed by the Kirby–Bauer disk diffusion test to study the drug-resistant pattern of the aforementioned organisms (Bauer et al., 1966; Clinical and Laboratory Standards Institute [CLSI], 2012).

#### *E*-Test

To determine the MIC of gentamicin for these isolates EzyMIC^TM^ Strip (GEN) (0.016–256 mcg/ml) and EzyMIC^TM^ Strip (HLG) (0.064–1024 mcg/ml) (HiMedia labs Pvt. Ltd.) were used. The test was performed on Mueller Hinton agar (HiMedia labs Pvt. Ltd.) swabbing a sterile cotton-swab soaked in inoculum prepared by comparing the turbidity of direct colony suspension to standard 0.5 McFarland. The EzyMIC^TM^ Strip were placed at the center of the agar plate and incubated at 37°C for 24 h. After incubation, the plates were read at the point of complete inhibition of all growth, including hazes, micro-colonies, and isolated colonies as indicated (Nachnani et al., 1992).

#### MIC of Gentamicin

To determine the MIC of gentamicin, broth dilution method was used according to standard broth microdilution method M07 A9 of Clinical and Laboratory Standards Institute [CLSI] (2012).

#### Checkerboard Assay

The stock solutions of gentamicin used ranged 2.5 to 160 μg/ml. The stock solutions of silver and zinc nanoparticles used ranged from 0.3 to 20 mg/ml. Double dilutions of both the antibiotic as well as the nanoparticles was used for the assay according to the recommendations of CLSI. A total of 50 μl of sterile nutrient broth was distributed aseptically in all the sterile microtiter plate (96-well plate). The antibiotic gentamicin was serially diluted along the abscissa, while the nanoparticle was diluted along the ordinate. An inoculum equal to 0.5 McFarland turbidity standards was prepared for each culture in sterile saline. Each well was inoculated with 10 μl of the culture. The microtiter plate was incubated at 37°C for 24 h. After incubation, the growth was observed by streaking a loopful from each well in sterile nutrient agar plates. The plates were incubated at 37°C for 24 h. The plates were observed for growth of the test organism. The combination of the drugs in which the growth is completely inhibited was considered as effective MIC for the combination (Breitinger, 2010).

The ΣFICs (fractional inhibitory concentrations) were calculated as follows:

∑FIC=FIC A+FIC B

where FIC A is the MIC of drug A (gentamicin) in the combination/MIC of drug A (gentamicin) alone, and FIC B is the MIC of drug B (nanoparticle) in the combination/MIC of drug B (nanoparticle) alone. The combination is considered synergistic when the ΣFIC is ≤0.5, indifferent when the ΣFIC is >0.5 to <2, and antagonistic when the ΣFIC is ≥2 (Rabadia et al., 2013). The overall study period was 12 months from material synthesis to performing all *in vitro* activities.

## Results

### Synthesis and Characterization of Nanoparticles

**FIGURE 1:**
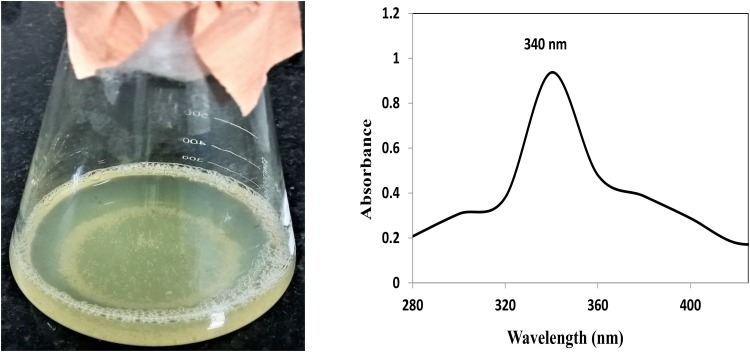
Typical precipitate formation and UV–Vis spectra of the synthesized zinc nanoparticles.

Synthesis of AgNP and ZnNP was achieved extracellularly using *P. hibiscicola* isolated from effluent of electroplating industry. Polydispersed silver nanoparticles of average 40 nm in size were obtained as mentioned in the previous work. In case of ZnNP, typical white precipitate formation as seen in **Figure 1** indicated the synthesis of zinc nanoparticles. The UV–Vis spectra (**Figure 1**) confirm the synthesis of zinc nanoparticles in the reaction mixture.

**FIGURE 2:**
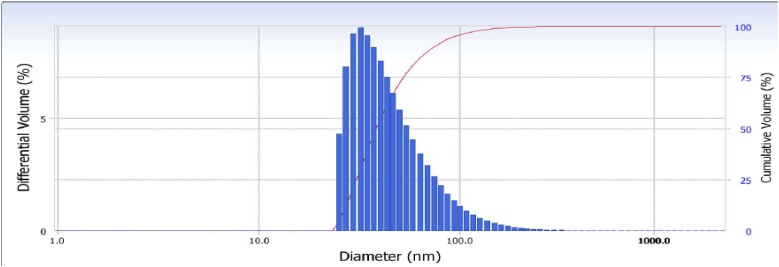
DLS of synthesized zinc nanoparticles.

Further characterization studies gave details about the nanoparticle size, morphology, surface charge, and crystalline nature. The dynamic light scattering (DLS) and zeta potential of the zinc nanoparticles (**Figure 2**) indicated synthesis of stable nanoparticles of size 110 nm and polydispersity index of −0.2. The zeta potential of the zinc nanoparticles was found to be 24.64 mV.

**FIGURE 3:**
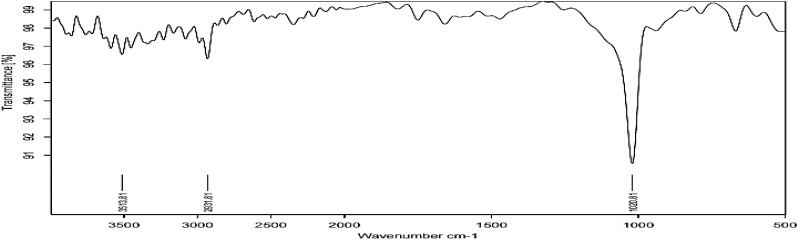
FTIR spectra of zinc nanoparticles.

**FIGURE 4:**
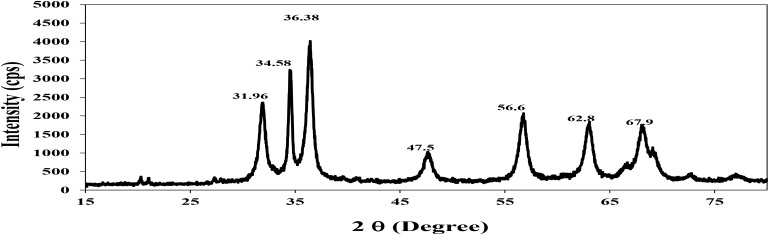
XRD pattern of zinc nanoparticles.

Fourier transform infrared **Figure 3** shows absorption bands at 3513, 2931, and 1020 cm^−1^ indicating the presence of capping agent with the nanoparticles. The spectra correspond to secondary alcohol, alkane, and amine regions. These functional groups have a role in stability/capping of nanoparticles. XRD pattern **Figure 4** shows 2𝜃 values at 31.96°, 34.58°, 36.38°, 47.5°, 56.6°, 62.8°, and 67.9°. All evident peaks could be indexed as the zinc oxide wurtzite structure (JCPDS Data Card No: 36-1451), which confirms the synthesized nanoparticles were free of impurities as it does not contain any characteristics XRD peaks other than zinc oxide peaks.

**FIGURE 5:**
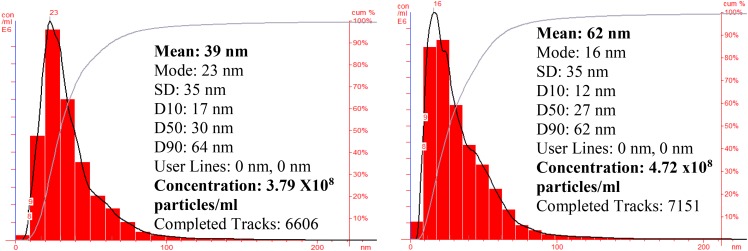
NTA of silver and zinc nanoparticles.

Nanoparticle tracking and analysis of AgNP and ZnNP (**Figure 5**) shows the particle size distribution which was in the range of 10–70 nm for silver nanoparticles while the mean size was found to be 39 nm with concentration of 3.79 × 10^8^ particles/ml; whereas for ZnNP, the particle size distribution was in the range of 5–90 nm, and mean size was 62 nm with concentration of 4.72 × 10^8^ particles/ml.

**FIGURE 6:**
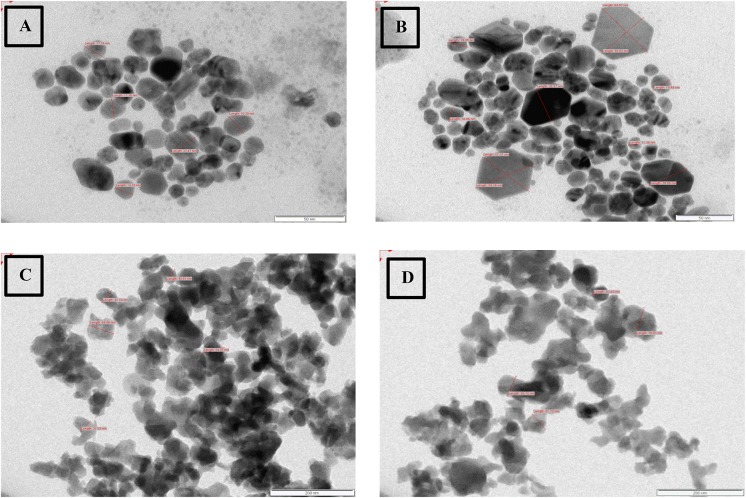
TEM of silver nanoparticles **(A,B)** and zinc nanoparticles **(C,D)**.

The size and morphology of silver nanoparticles synthesized were observed in TEM with average size of 40 nm and most of the population consisting of single crystalline nanoparticles. Spherical zinc nanoparticles of varying size were observed with average size of 60 nm (**Figure 6**).

### Toxicity of Nanoparticles

**FIGURE 7:**
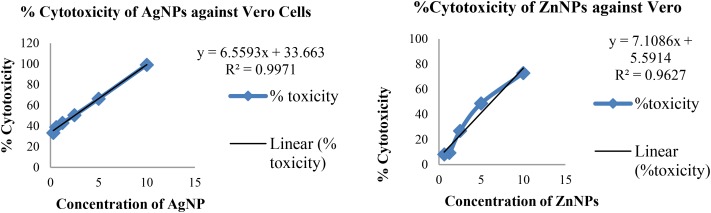
Plot of cytotoxicity vs concentration of nanoparticles.

Cytotoxicity was evaluated in the range of 0.3 to 10 mg/ml concentration of nanoparticles. Toxicity of a compound is calculated by determining the IC_50_ value, which is that concentration at which growth of cells is inhibited by 50%. The percentage cytotoxicity is plotted against the concentration of nanoparticle as seen in **Figure 7**.

Based on toxicity of nanoparticles to Vero cell line, the IC_50_ values for silver and zinc nanoparticles were found to be 5.54 and 6.24 mg/ml, respectively.

### Antimicrobial Activity of Nanoparticles

#### Minimum Inhibitory Concentration

Antimicrobial activity was assessed up to 10 mg/ml concentration of nanoparticle, which is almost double the concentration of cell toxicity to normal cells. For therapeutic consideration as an antimicrobial for synthesized nanoparticles only activity obtained at 5 mg/ml or below was considered. The MIC of both nanoparticles against range pathogenic bacterial strains is as mentioned in **Table 1**.

**Table 1:** MIC of nanoparticles against range of pathogenic bacteria.

Sr. No.	Organism	ATCC No.	MIC mg/ml	
			AgNPs	ZnNPs
**Gram-Negative Bacteria**				
1.	*E. coli*	25922	2.5	10
2.	*E. aerogenes*	13048	0.6	>10
3.	*K. pneumoniae* ( ESBL)	700603	2.5	>10
4.	*K. pneumoniae*	10031	5	10
5.	*S. typhimurium*	14028	2.5	5
6.	*S. abony*	6017	2.5	>10
7.	*S. boydii*	8700	2.5	>10
8.	*P. mirabilis*	21100	10	1.25
9.	*P. aeruginosa*	27853	2.5	>10
**Gram-Positive Bacteria**				
10.	*S. aureus*	25923	5	2.5
11.	*S. aureus*	6538	5	5
12.	*S. aureus* (MRSA)	43300	2.5	2.5
13.	*S. epidermidis*	12228	5	5
14.	*E. faecalis* (VRE)	51299	10	>10
15.	*B. subtilis*	6633	>10	>10
16.	*B. cereus*	11778	5	10

#### Anti-mycobacterial Activity – Broth Dilution Method

**Table 2** shows the MICs obtained for standard strain and MDR strain of *M. tuberculosis* against both nanoparticles.

**Table 2:** MIC of nanoparticles against standard and MDR strain of *Mycobacterium tuberculosis.*

Sr. No.	Organism	MIC (mg/ml)	
		AgNPs	ZnNPs
1.	*M. tuberculosis* (H37RV)	1.25	1.25
2.	*M. tuberculosis* (MDR – Clinical isolate)	1.25	1.25

### Synergistic Activity of Gentamicin and Nanoparticles

Nanoparticles were estimated for its synergistic action with gentamicin against MDR strains. For which susceptibility and MIC of gentamicin were checked, and results of that were as summarized in **Tables 3**, 4. Representative images in the **Supplementary Material**.

**Table 3:** Susceptibility for gentamicin.

Sr. No.	Organism	Diameter of Zone of Inhibition (mm)	
		Gentamicin (10 μg)	High-Level Gentamicin (120 μg)
1.	*S. aureus* ATCC 43300 (MRSA)	6 (R)	12 (R)
2.	*K. pneumoniae* ATCC 700603 (ESBL)	18 (S)	24 (S)
3.	*P. aeruginosa* ATCC 27853	25 (R)	32 (R)
4.	*E. faecalis* ATCC 51299 (VRE)	6 (R)	6 (R)
5.	*P. aeruginosa* (MDR) Clinical isolate	6 (R)	6 (R)

*R, resistant; S, susceptible (Clinical and Laboratory Standards Institute [CLSI], 2014).*

**Table 4:** *E*-test MIC for gentamicin.

Sr. No.	Organism	EZY-MIC	
		Gentamicin (0.016–256 mg)	High-Level Gentamicin (0.064–1024 mg)
1.	*S. aureus* ATCC 43300 (MRSA)	256 (R)	1024 (R)
2.	*K. pneumoniae* ATCC 700603 (ESBL)	3 (S)	4 (S)
3.	*P. aeruginosa* ATCC 27853	1 (S)	1.5 (S)
4.	*E. faecalis* ATCC 51299 (VRE)	256 (R)	1024 (R)
5.	*P. aeruginosa* (MDR) clinical isolate	256 (R)	1024 (R)

*R, resistant; S, susceptible (Clinical and Laboratory Standards Institute [CLSI], 2014).*

#### MIC of Gentamicin

The MIC of gentamicin by microbroth dilution method was evaluated up to 320 μg/ml, considering the limit and toxicity of gentamicin. **Table 5** shows the MICs obtained for each test strain.

**Table 5:** MIC for gentamicin – microbroth dilution method.

Sr. No.	Organism	MIC of Gentamicin (μg/ml)
1.	*S. aureus* ATCC 43300 (MRSA)	20
2.	*K. pneumoniae* ATCC 700603 (ESBL)	2.5
3.	*P. aeruginosa* ATCC 27853	<0.6
4.	*E. faecalis* ATCC 51299 (VRE)	>320
5.	*P. aeruginosa* (MDR) clinical isolate	>320

#### Checkerboard Assay

For determination of synergistic activity of synthesized nanoparticles with gentamicin against test strains checkerboard assay was performed. The FIC values and interpretation of synergism are as shown in **Table 6**.

**Table 6:** Synergistic activity of nanoparticles and gentamicin.

Sr. No.	Organism	ΣFIC Value	Result
1.	*S. aureus* ATCC 43300 (MRSA)	0.5	Synergistic (with AgNPs)
2.	*K. pneumoniae* ATCC 700603 (ESBL)	0.3	Synergistic (with AgNPs)
3.	*P. aeruginosa* (MDR) clinical isolate	0.2–0.5	Synergistic (with AgNPs)
4.	*S. aureus* ATCC 43300 (MRSA)	0.5	Synergistic (with ZnNPs)

*AgNPS, silver nanoparticles; ZnNPs, zinc nanoparticles.*

## Discussion

Bacterial synthesis of silver nanoparticles by *P. hibiscicola* was first reported in our previous work (Punjabi et al., 2017). Silver nanoparticles synthesized were characterized by various spectral and microscopic techniques to specifically confirm the synthesis and determine the details of synthesized nanoparticles.

In this study, the NTA revealed the average size of AgNP ranged 10 to 70 nm, but the mean size of synthesized silver nanoparticles was found to be 39 nm. This is in accordance to similar studies with biosynthesis of nanoparticles and its measurement by NTA. Birla et al. (2013) report the size distribution of nanoparticles obtained on a particle-by-particle basis in case of NTA which enables particle population, size, intensity of nanoparticle in suspension, and simultaneously determining their Brownian motion. The NTA calculates the particles size by distance traveled by them, and calculation is based on Stokes–Einstein equation. The average size distribution of silver nanoparticles synthesized by *Fusarium oxysporum* in their study was 41 nm (Birla et al., 2013). TEM revealed an average size of 40.0 nm, which is in accordance with results obtained by NTA. The TEM analysis indicated that the particles were crystalline in nature.

The extracellular biosynthesis of silver and zinc oxide nanoparticles was investigated using *Pichia fermentans* JA2 by Chauhan et al. (2015). The biosynthesized silver nanoparticles were found to inhibit most Gram-negative clinical pathogens used in the study, while zinc oxide nanoparticles only inhibited *P. aeruginosa*. The silver and zinc oxide nanoparticles synthesized using *Pichia fermentans* JA2 had potent antibacterial effect that leads to notable contribution to pharmaceutical associations.

The above report boosts the idea of synthesizing zinc nanoparticles by *P. hibiscicola*. Thus, studies were undertaken to synthesize, optimize, and characterize zinc nanoparticles, which were found to be of variable shapes and the average size of 60 nm. Although DLS reports 110 nm, NTA shows 62 nm final size of nanoparticles was only considered as obtained in TEM micrographs, which were 60 nm. In most case, synthesis of zinc oxide nanoparticles often takes place which could highly be possible in this case as well. This could be alleged on basis of the spectra in UV–Vis spectrophotometer and XRD pattern, which does indicate the presence of oxide specific peaks. Different functional groups identified as secondary alcohols, amines, and alkanes by FTIR analysis, which could mainly be due to biological synthesis.

Jayaseelan et al. (2012) present a work for biosynthesis of zinc oxide nanoparticles (ZnONPs) using bacteria, *Aeromonas hydrophila*. The ZnO NPs were characterized, and a peak at 374 nm in the UV–vis spectrum was observed. Further, XRD confirmed the crystalline nature of the nanoparticles and AFM exhibited the morphology of the nanoparticle to be spherical, oval with an average size of 57.72 nm. The FTIR peaks summarized as alkynes, alkanes, alkenes, primary amines, alcohols, acetate, ethers, and carboxyl compounds. The results of zinc nanoparticles synthesized by *P. hibiscicola* are highly compatible with this study. It could well be said so that synthesis of zinc oxide nanoparticles formation takes place by *P. hibiscicola* also.

The most vital parameter to determine the therapeutic feasibility is toxicity of the material. Therefore, *in vitro* cytotoxicity of both nanoparticles synthesized were assessed on animal tissue. The technique employed to do so was MTT assay, which is a colorimetric estimation of living cells determined by absorbance values upon treatment with the drug for a stipulated amount of time. The cell line used to evaluate the toxicity of nanoparticles was Vero a non-cancerous epithelial cell line commonly used for the said purpose.

In one of the reports, use of AgNP as a therapeutic agent is limited due to its cytotoxicity against mammalian cells. Wherein cytotoxicity of *P. putida* synthesized silver nanoparticles against HEp-2 cells was determined by MTT assay. No significant cytotoxic effect at 25 μg/ml concentration was found, which was lethal for the bacteria tested for antibacterial efficacy (Gopinath et al., 2017). This study highlights and supports the results obtained for nanoparticles synthesized by *P. hibiscicola* although the range of concentration varies mainly due to the presence of protein in the nanomaterial. The purification of nanoparticles was not carried out and freeze-dried powders of as-synthesized nanoparticle were used for cytotoxicity and other activities, leading to the larger amount of material.

One of the most important ailments affecting thousands around the world is microbial infections and more to concern is its drug resistance happening at a rapid pace. Special emphasis needs to be given to drug-resistant infections of bacteria in the community and hospital-acquired infections like ESBL, MRSA, VRE, MDR, *P. aeruginosa* infections, and MDR *M. tuberculosis* infections. The resistance development has been concerning, and world organizations have been raising alarms at regular intervals. This has also lead to screening a vast number of agents from both biological as well as the chemical cluster for antimicrobial potential. Nanoparticles have potentially all the requisites for qualification as an antimicrobial agent. Not only can nanoparticles have efficiency against this infection causing etiological agents but can also prevent the current therapeutic agents from being rendered useless due to resistance, by alternative or supplementary therapies with nanoparticles along with regular antibiotics. Thus, an interesting aspect was to evaluate nanoparticles synthesized by *P. hibiscicola* for its antimicrobial activity.

Only activity obtained at or below 5 mg/ml was considered suitable for antimicrobial potential against the test strain considering the toxicity of nanoparticles at higher concentrations. Zinc nanoparticle gave activity only against two Gram-negative and four Gram-positive strains, importantly against MRSA at 2.5 mg/ml. Silver nanoparticles were the most potent antimicrobial with activity against most bacteria. In case of the drug-resistant variants for both ESBL and MRSA, activity was observed at 2.5 mg/ml. Both AgNP and ZnNP had anti-TB activity at 1.25 mg/ml for both strains. These results definitely imply that nanoparticles synthesized by *P. hibiscicola* have great antimicrobial potential. This is encouraging to do further evaluations and assess its practical application with more specific *in vivo* studies.

Abd-Elnaby et al. (2016) reported an excellent antibacterial activity against Gram-negative and Gram-positive bacterial pathogens by microbiologically synthesized AgNPs. Mostafa et al. (2015) report activity of chemically synthesized silver nanoparticles against *P. aeruginosa*, *S. aureus*, *Streptococcus pyogenes*, and *S. typhi.* A report stating anti-tuberculosis activity of biosynthesized silver nanoparticles-mentioned spherical-shaped silver nanoparticles with the average particle size of 5 nm, had anti-mycobacterial activity against *M. tuberculosis* and clinical isolates of multi-drug resistant *M. tuberculosis* (MDR-TB) (Banu and Rathod, 2013).

Arrays of pathogens causing different infections were inhibited with the synthesized nanoparticles suggesting its efficiency in controlling infections. This also recommends for assessment of these nanoparticles for synergistic activity with common antibiotics. Thus, synergistic activity against drug-resistant strains was evaluated with antibiotic gentamicin. Gentamicin is an aminoglycoside and commonly used antibiotic against both Gram-positive and Gram-negative bacteria. Currently, gentamicin is susceptible to some and resistant to some bacterial isolates, but it could fast be completely resistant if measures to control resistance are not in place soon. Thus, hypothetically nanoparticles could rescue this situation with its potential synergistic efficiency if found.

Antibacterial susceptibility against gentamicin was assessed by disk diffusion method followed by MIC of gentamicin by *E* – test and micro broth dilution method. The results of disk diffusion and *E*-test identify all the isolates as resistant to gentamicin as per CLSI guidelines of 2014. The MIC of gentamicin by micro broth dilution method was evaluated with a maximum concentration of 320 μg/ml, which is reportedly a toxic concentration. Synergistic activity of nanoparticles and gentamicin was evaluated by Checkerboard method, considering the MICs and toxicity of both gentamicin and nanoparticles.

Promising results were obtained for synergistic activity, zinc nanoparticles exhibited synergism with gentamicin in case of MRSA, so did silver nanoparticles for MRSA, ESBL, and MDR *P. aeruginosa*. A study on the fugal-mediated synthesis of silver nanoparticles reports synergistic activity of silver nanoparticles with amikacin, kanamycin, and streptomycin against *E. coli*, *S. aureus*, and *P. aeruginosa.* The report concluded synergistic action of antimicrobial agent can significantly curtail side effects by reducing dosages. Thus, use of nanoparticles combined with antibiotics can improve their efficacy against various pathogenic microbes (Barapatre et al., 2016). The results of synergistic activity of nanoparticles synthesized by *P. hibiscicola* are in accordance with this report and rightly suggested nanoparticles can indeed be one of the most effective agents in combating serious infections that are rendered untreatable with available antibiotics. There are limited studies with this extent of antimicrobial activity, with qualitative as well as quantitative data against wide range of ATCC and clinical strains. Further to this, determining the mode of action will be an important aspect. But it is well known that most metallic nanoparticles cause oxidative damage to microbes due to excessive generation of reactive oxygen species (ROS), this can be extended to the nanoparticles synthesized.

The extensive potential of silver and zinc nanoparticles against drug-resistant strains of community and hospital-acquired infections opens a new arena of antimicrobials for treatment, supplementary prophylaxis, and prevention therapy. Biologically synthesized nanoparticles can become future anti-infectives and prove to be one of the most potential therapeutic agents.

## Conclusion

This study covers certain major areas of nanoscience like biological synthesis, characterization, and biomedical application. It provides an extremely potential isolate capable of synthesizing silver and zinc nanoparticles. Characterization studies revealed synthesis of stable nanoparticles of average size 50 nm for silver and 60 nm for zinc nanoparticles. The cytotoxicity studies of nanoparticles against normal cells (non – cancerous) revealed the toxic concentrations for both. Toxicity is the most important factor for therapeutic application. Efficiency of these nanoparticles against drug-resistant pathogen makes it a viable candidate for therapeutics. Thus, biologically synthesized nanoparticles can become future anti-infective agents and can be formulated for drugs or topical agents.

## Author Contributions

KP conceptualized and executed the entire experiment as well as the writing of this work. SM helped with the characterization studies, mainly the XRD analysis. RC, VC, and DD helped with the experimental work, including microbial assays and toxicity analysis. VC also helped write and format the manuscript. SD managed the successful completion of the entire project and played an instrumental role in the manuscript.

## Conflict of Interest Statement

The authors declare that the research was conducted in the absence of any commercial or financial relationships that could be construed as a potential conflict of interest.
