# Supplementary material for: Efficiency of Biosynthesized Silver and Zinc Nanoparticles Against Multi-Drug Resistant Pathogens
Source: Front Microbiol. 2018 Sep 20;9:2207. doi: 10.3389/fmicb.2018.02207 (PMC6159740; doi:10.3389/fmicb.2018.02207)
Supplement: Supplementary file 1 [file Data_Sheet_1.DOCX]

Disk diffusion test to determine the drug susceptibility against Gentamicin for test strains


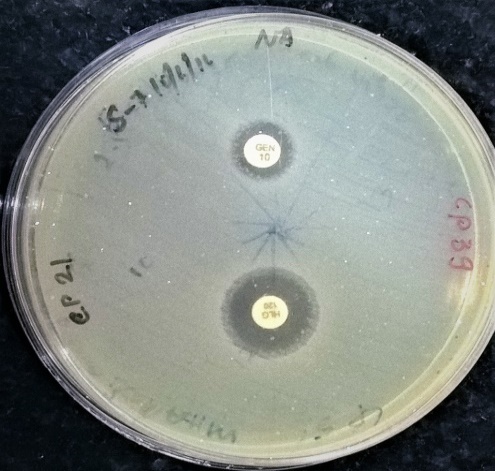

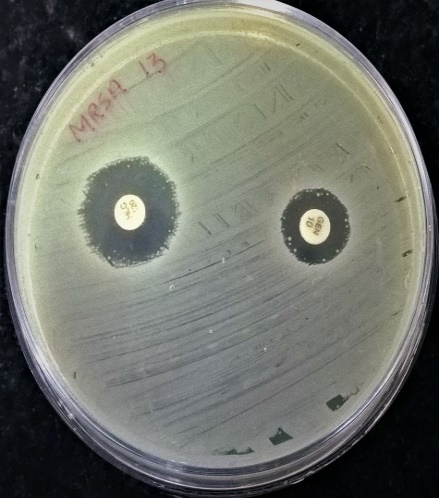

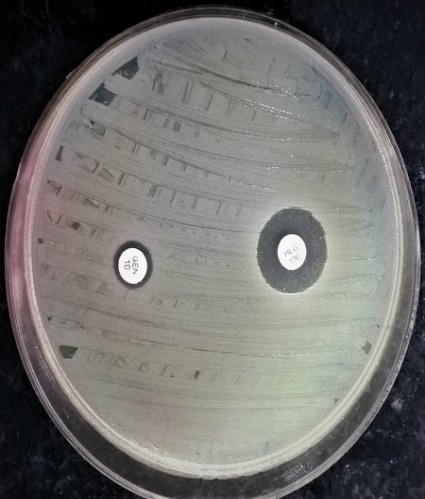


G

*E. coli* (Clinical isolate)

MRSA (Clinical isolate)

*K. pneumoniae* (Clinical isolate)

HLG

HLG

HLG

G

G

**G – Gentamicin; HLG – High Level Gentamicin**

(E Test) EZYMIC of test strains against Gentamicin and High level Gentamicin


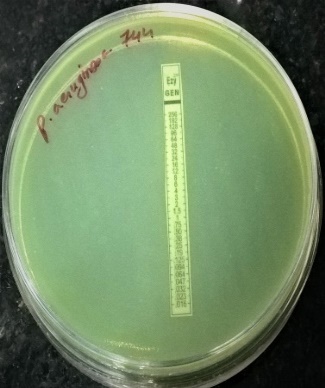

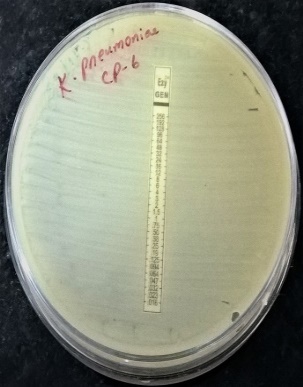

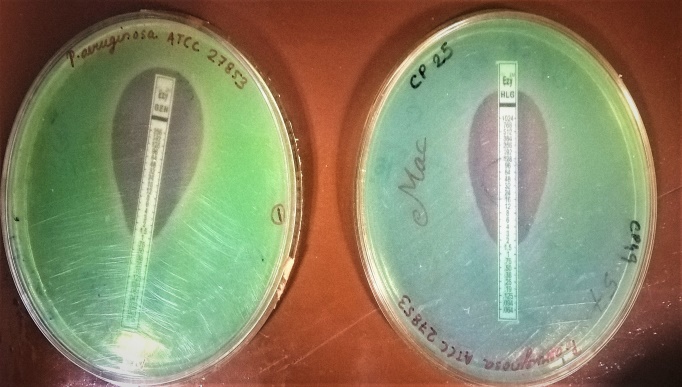


*P. aeruginosa*

(ATCC)

HLG

GEN

GEN

GEN

*P. aeruginosa*

(Clinical isolate)

*K. pneumoniae*

(Clinical isolate)

**GEN – E strip of Gentamicin; HLG – E strip of High Level Gentamicin**
